# Toward Self‐Powered Wearable Adhesive Skin Patch with Bendable Microneedle Array for Transdermal Drug Delivery

**DOI:** 10.1002/advs.201500441

**Published:** 2016-04-19

**Authors:** Hao Wang, Giorgia Pastorin, Chengkuo Lee

**Affiliations:** ^1^ Department of Electrical and Computer EngineeringNational University of Singapore4 Engineering Drive 3Singapore117576Singapore; ^2^ Center for Sensors and MEMSNational University of Singapore4 Engineering Drive 3Singapore117576Singapore; ^3^ Singapore Institute for Neurotechnology (SiNAPSE)National University of Singapore28 Medical Drive, #05‐CORSingapore117456Singapore; ^4^ NUS Suzhou Research Institute (NUSRI)Suzhou Industrial ParkSuzhou215123P. R. China; ^5^ Pharmacy Department National University of SingaporeSingapore117543Singapore; ^6^ NanoCoreFaculty of EngineeringNational University of SingaporeSingapore117576Singapore; ^7^ NUS Graduate School for Integrative Sciences and EngineeringCentre for Life Sciences (CeLS)Singapore117456Singapore

**Keywords:** bendable microneedle, self‐powered, wearable adhesive skin patch

## Abstract

**A wearable adhesive skin patch for transdermal drug delivery** is developed with bendable microneedles, dry adhesive and triboelectric energy harvester (TEH). The bendable microneedle array can overcome the needle breakage issue. The dry adhesive can realize a conformal attachment. The TEH can generate power when attached on flat skin or joint to power active components to be integrated in the future.

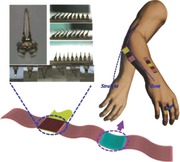

Wearable flexible electronic medical devices have received major attention recently owing to their considerable practicability for several applications,^1^, ^2^, ^3^, ^4^, ^5^, ^6^, ^7^, ^8^, ^9^, ^10^ including health monitoring and drug delivery for disease treatment.^11^, ^12^, ^13^ The traditional drug delivery method using hypodermic needles may be an unpleasant process to many patients. Thus, microneedle‐based transdermal drug delivery approaches have been investigated by changing different kinds of materials and configurations.^14^, ^15^, ^16^, ^17^, ^18^, ^19^, ^20^, ^21^, ^22^, ^23^, ^24^, ^25^, ^26^, ^27^, ^28^, ^29^, ^30^, ^31^, ^32^, ^33^, ^34^, ^35^, ^36^, ^37^, ^38^, ^39^, ^40^, ^41^

Currently the safety issue caused by needle breakage after skin penetration step is the main concern for clinically relevant applications. High aspect ratio and sharp tips made of rigid materials are necessary for conventional microneedles to provide a successful and reliable skin penetration.^16^, ^25^, ^26^ Normally, microneedles made of materials of high Young's modulus such as nickel,^12^, ^38^ stainless steel,^26^, ^42^ and silicon,^14^, ^25^ can avoid this needle breakage. However, these materials lack of biocompatibility, which is a key requirement for medical devices. On the other hand, microneedles made of biocompatible polymers or natural fibers suffer from low mechanical strength. The consequent needle breakage can be induced either by very high buckling force attributed to the deformable skin surface during the skin penetration or by the lateral movement between the microneedle patch and the skin surface during the drug administration. Here we propose a novel drug delivery skin patch using an array of bendable microneedles. This bendable microneedle array with a soft base and a rigid sharp tip can tolerate the deformation associated with skin stretching without breakage when the skin patch is applied on the joint such as elbow and knuckle for osteoporosis treatment. In other cases, such as diabetes, for which microneedle patches are normally applied on the arm or abdomen, a lateral movement between the microneedle patch and skin surface may occur due to either the occasional touch or friction. In such case, the bendable microneedle will be dragged out of the skin instead of leaving a broken needle in the skin when lateral movement occurs. The sharp tips assembled onto the soft bases can be either non bio‐dissolvable, i.e., made of SU‐8,^43^ or biodissolvable, i.e., made of maltose.^44^ For the configuration with SU‐8 sharp tips, the outlets of the microneedles, connecting with the drug reservoirs, are always exposed to air. Both water‐soluble and lipophilic drugs can be immediately delivered just after skin penetration. For the configuration with maltose sharp tips, the outlets can be fully encapsulated by maltose to inhibit the solvent evaporation of lipophilic drug formulation.^45^ The drug can be delivered when the maltose tips are melted after skin penetration. However, the microneedle with maltose tip is not suitable for application of water‐soluble drug formulation.^46^The evaporation of water in solvent will cause maltose tip melting. Therefore, the water‐soluble drug formulation can only be stored in patches with SU‐8 sharp tips.

Another key feature for wearable medical device is the fixation method. Acrylic medical bandage is widely used for medical patches. However, there are increasing demands on less‐irritating, biocompatible medical bondages, as aging skin is more sensitive and vulnerable to a prolonged exposure, e.g., insulin delivery, as in the case of conventional medical patches. Dry adhesive, which is inspired by the hierarchical structure on Gecko foot hair,^43^, ^44^ possesses several advantages compared with conventional acrylic medical bandages: First, it shows repeatable and restorable adhesion with surface cleaning after each usage. Second, the physical structure to generate adhesive force is less affected by surface contamination, oxidation and other environmental stimuli. Third, the space between the pillars for ventilation of air should provide better bio‐compatibility. Hence, we also adopt the dry adhesive for the fixation of the whole device on the skin.

To construct a complete drug delivery system for home healthcare monitoring, it is important to have feedback control function of the delivered drug. It can alert patients and provide the guidance when the dose of the drug to be delivered should be accurately controlled.^47^, ^48^, ^49^, ^50^ Currently, several wearable sensors for health monitoring have been explored, including devices that measure hydration,^51^ strain,^52^ glycaemia,^53^, ^54^, ^55^ metabolic acid,^56^ and cardiorespiratory signals.^53^ For a further integration of diversified sensors mentioned above and construct a standalone wearable drug delivery skin patch with capability of health monitoring, signal process and interfacing with external cloud computing apparatus, a build‐in energy source is inevitable to power components such as integrated circuits(ICs), microprocessor, liquid‐crystal display(LCD) reading panel. Triboelectric energy harvester (TEH) typically using patterned PDMS is compatible with the fabrication process of flexible skin patch with microneedles. Thus, TEH is the most promising technique to fulfil the above‐mentioned requirements. In this paper, we leverage the same microstructure of the dry adhesive for triboelectric patch to harvest energy from the contact with human skin, which has not been reported yet. Considering the locations for patch attached can be on flat skin surface like arm and abdomen or on joints like elbow and knuckle in different applications, we studied and developed two kinds of configurations to adapt these two situations for energy harvesting.

We propose a stretchable adhesive flexible microneedle skin patch attached onto flat skin surface or joint parts, i.e., elbow and knuckle, as shown in **Scheme** **1**a. The whole skin patch consists of four functional components: bendable microneedle patch; dry adhesive patch; triboelectric energy harvester patch and drug delivery system with pump and drug reservoirs connected to the bendable microneedle skin patch. The detailed structure is depicted in Scheme 1b. The microneedle and triboelectric patches are connected with three dry adhesive patches to make the whole wearable device able to be fixed onto the curved skin surface. The microfluidic control system with pump and drug reservoirs^57^ can be assembled at the backside of the microneedle patch to control the drug delivery after the skin penetration. The detailed working principle of the pump system is provided in Figure S3 (Supporting Information).

**Scheme 1:**
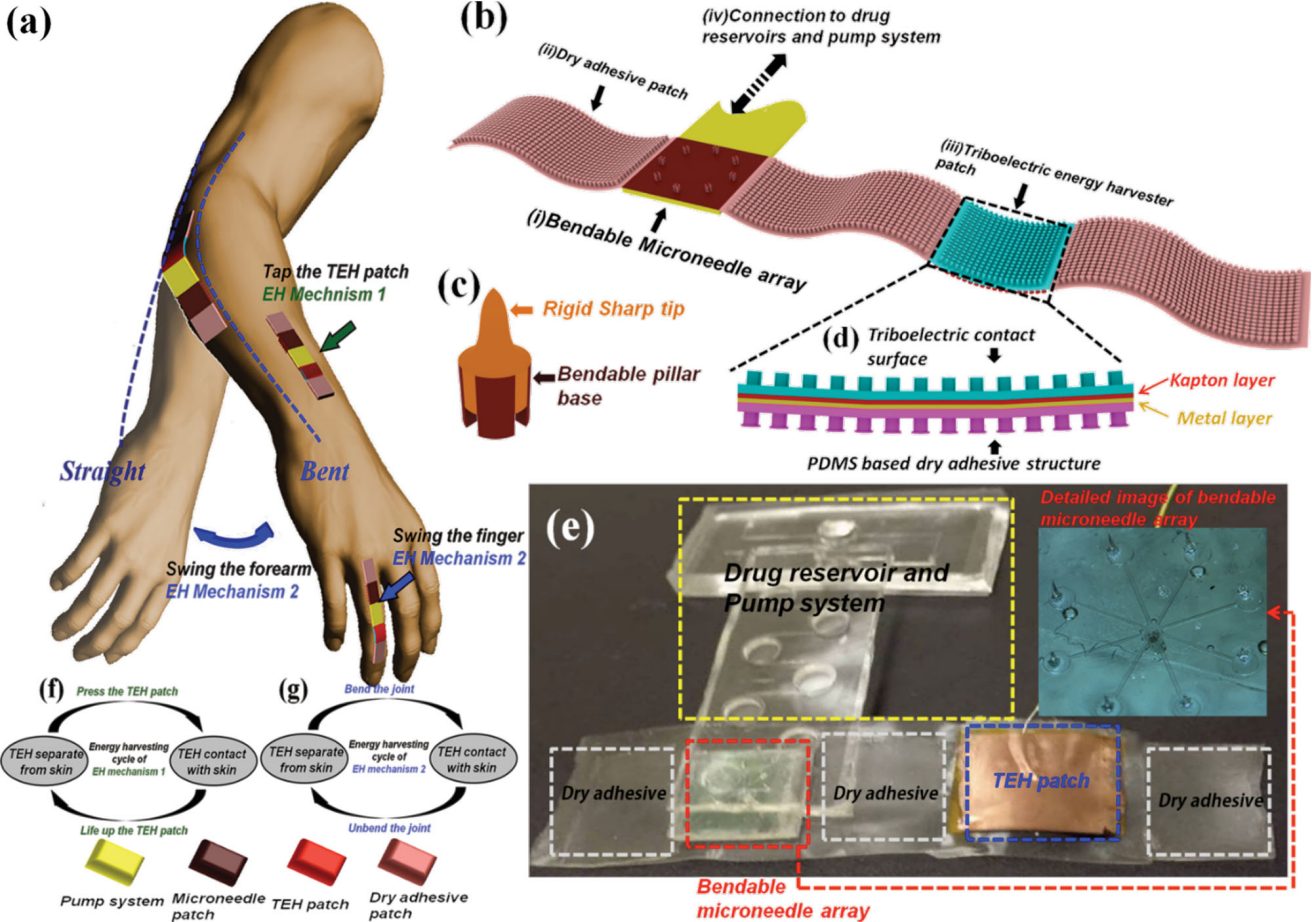
a) Concept of the flexible microneedle skin patch attached on arm, elbow, and knuckle. The patch consists of four functional components integrated on a whole PDMS sheet: Microneedle patch; Dry adhesive patch, TEH patch, and pump system. b) Detailed structure and functional components of the flexible microneedle skin patch; c) Detailed structure of an individual bendable microneedle; d) Detailed layer structure of the TEH patch; e) Image of fabricated skin patch; f) Attach the patch on flat skin surface like arm, power can be generated by pressing and lifting up the TEH patch g) Attach the patch at the joint like elbow or finger, power can be generated by bending and unbending the elbow or finger.

The structure of an individual bendable microneedle is shown in Scheme 1c where it consists of a bendable four‐beam‐pillar base and a rigid sharp tip. The bendable pillar base is made of PDMS with optimized stiffness to ensure a high success rate of skin penetration, while certain volume deformation is allowed. A rigid sharp tip for skin penetration can be assembled onto the four‐beam‐pillar base structure by using double‐drawing lithography process.^58^, ^59^ The gaps between the pillars can be partially filled with the same materials to form microneedles during the drawing lithography step. It provides anchoring between the sharp tip and soft base in order to fix the sharp tip onto the soft base and protect it from breakage when the whole microneedle is bent. Another functional component is the TEH patch for energy harvester. The triboelectric contact surface with PDMS micropatterned structure can enhance the performance. Here, we used the micropillar array with and without mushroom top, which is obtained from the same fabrication process of the dry adhesive patch, as the surface micro structure for TEH patch. Meanwhile, we also tested the sample with pyramid micro structure, which is normally used for THE,^60^, ^61^, ^62^, ^63^, ^64^, ^65^, ^66^ as a comparison.

Two methods were developed to generate power from the TEH patch by applying the skin patch on different locations of the human body. When the patch is attached to the elbow of a straight forearm, the spacing between two dry adhesive patches is slightly shorter than the length of the TEH patch. Thus the TEH patch is bent and has no contact with skin surface at this initial state. Then when the joint is bent, the TEH patch is stretched and in contact with the skin. Thereafter, when the joint is straight again, the spacing between two dry adhesive patches is compressed to make the TEH bent and separate from skin. Power can be harvested by repeating this cycle as illustrated in Scheme 1g.

For the case in which the patch is applied onto a flat skin surface like arm and abdomen, power can be generated by pressing and releasing the TEH patch to induce contact and separation between TEH patch and skin surface as illustrated in Scheme 1f. However, due to the sticky surface of PDMS, once the triboelectric contact surface is pressed onto skin, it cannot automatically separate from skin when the pressing is released. To solve this problem, a dry adhesive patch is assembled at the backside of the TEH patch as shown in Scheme 1d. When the finger lifts up, the dry adhesive can provide a pulling force to make the TEH detach from the skin surface. Because the adhesive force provided by the dry adhesive is limited, the dry adhesive will detach from finger when lifted up to a certain height. In order to have a maximized output power of TEH patch, the dry adhesive is optimized to provide a maximum lift‐up height.

In order to solve the needle breakage issue after skin penetration, a unique bendable microneedle design is proposed here. The bendable microneedle consists of two parts: a soft four‐beam‐pillar base made of PDMS and a stiff SU‐8 sharp tip. The material of the sharp tips can be either SU‐8 (**Figure** **1**a) or maltose (Figure 1b). These sharp tips were assembled onto the four‐beam‐pillar structured array, which has four vertical gaps along the sidewalls (Figure 1a2 and Figure 1b2). These four vertical gaps serve two functions: first, because the sharp tips made of SU‐8 are not biodissolvable, the drug could be delivered out through these gaps; second, during the drawing lithography process, the sharp tips could form an anchor shape in these gaps to enhance the adhesion between sharp tips and four‐beam‐pillar base. Due to the flexibility of the PDMS pillars, the needle will bend when the lateral force applied onto the microneedle exceeds the threshold. These anchors of the rigid sharp tips in the gaps could fix the sharp tips onto the PDMS pillar and protect them from detaching the PDMS pillars when the whole microneedle is bent. Figure 1a3 and Figure 1b3 show the demonstrations of the bendable needle when a glass slide pushes the microneedle array from a lateral direction. These needles will bend when the lateral force is applied. They will recover to the initial state as long as the force is removed. After skin penetration, the relative movement between the microneedle patch and skin is inevitable during the manual operation, which is one the major reasons for needle breakage. The soft PDMS pillars at the bottom of the microneedles will bend to absorb the mechanical strain caused by lateral movement and further prevent the breaking when this relative movement occurs. Figure 1a4 shows a demonstration when the relative movement between skin and microneedle occurs after skin penetration. The dash line indicates the contour profile of needle position inside skin; from (a4‐i) to (a4‐iii), the needle penetrated the skin, the whole sharp tip was into the skin; from (a4‐iv) to (a4‐v), a lateral movement occurred, the needle had a displacement from its original position. The PDMS pillars beneath the SU‐8 tip bended and the SU‐8 tip still remained within the skin (a4‐vi); the needle was taken out of the skin and recovered to its original shape without breakage. This demonstration confirms that when the relative movement occurs, the needles will bend to match the new position of the skin. If the relative movement is too large, then the needles will be dragged out the skin and recover the initial state.

**Figure 1:**
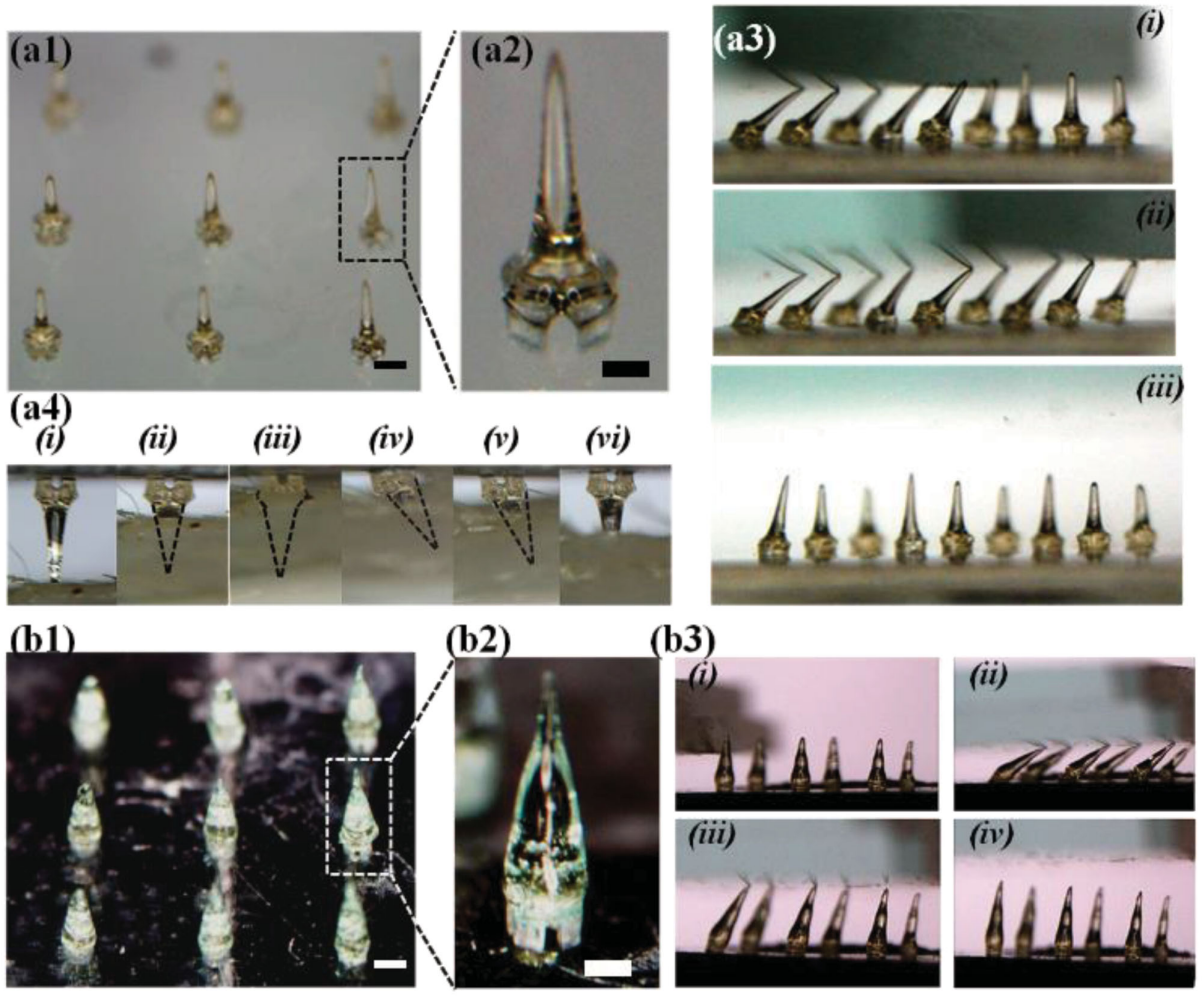
Demonstration of the bendable microneedle array. a1) Optical image of the microneedle array with SU‐8 sharp tips, the scale bar is 300 μm; a2) Detailed optical image of an individual bendable microneedle with SU‐8 sharp tip, the scale bar is 150 μm; a3) Demonstration of the bendable microneedle array with SU‐8 sharp tips when an lateral force is applied; i) A glass slide began pushing the needle array from a lateral direction, part of the needle array bended; ii) All the needle array was pushed by the glass slide and bended; iii) The glass slide was removed, all the needles recovered to their original shape without breakage; a4) Demonstration of the bendable microneedle when relative movement between skin and microneedle occurs after skin penetration. The dash line is used to indicate the contour profile of needle position inside skin; i) A bendable needle is out of skin before the penetration; ii) The microneedle pressed to penetrated the skin; iii) The whole needle is into the skin; iv) Al lateral movement occurs, the skin moves rightwards. The need is bent to adapt the lateral movement; v) The distance of the lateral movement exceeds the threshold of the bendable needle, then the microneedle is out of the penetration hole; vi) Lower the skin sample and make the bendable microneedle separate from skin surface. The needle recovers to its initial shape; b1) Optical image of the microneedle array with Maltose sharp tips, the scale bar is 300 μm; b2) Detailed optical image of an individual bendable microneedle with maltose sharp tip, the scale bar is 150 μm; b3) Demonstration of the bendable microneedle array with maltose sharp tips when a lateral force is applied. The process is similar as in (a3).

Due to the elasticity of PDMS, the bottom part of the microneedles, which is represented by the PDMS pillars, will bend when the force applied onto the microneedles exceeds the buckling force. To realize a successful skin penetration, the stiffness of the PDMS pillars are expected to be as high as possible. Thus, we conducted a study of the stiffness of the PDMS by tuning the mix ratio of elastomer and curing agent. The PDMS with higher concentration of curing agent can have a higher stiffness. Here we tested the samples with mix a ratio of 1:4, 1:6, 1:8, and 1:10 for microneedles with both SU‐8 and maltose sharp tips. Individual microneedles with PDMS base and SU‐8 sharp tips were subjected to load in order to study their mechanical stability. The variation of measured bending force versus displacement was recorded. **Figure** **2**a shows the measured curve of one sample. There are three parts: non‐contact region, contact region, and bend point, as indicated in this curve. The sharp drop of the force after the bend point confirms that the needles will not bend when the applied force is below the threshold. The forces of bend points of all the samples with different mix ratio of PDMS are shown in Figure 2b. For each mix ratio, ten needles were tested. As shown in Figure 2b, for the mix ratio of 1:4, the bending force is about 0.8 N for SU‐8 sharp tips and 1.14 N for maltose sharp tips. The bend force decreases with the decrease of the mix ratio. The buckling force of needle with maltose sharp tip is higher than that of the needle with SU‐8 sharp tip because of the different shapes of the sharp tips. As shown in Figure 1a2 and b2, the SU‐8 sharp tip is slimmer than the maltose needle. This is because the viscosity of SU‐8 is lower than that of maltose. During the drawing lithography process, the SU‐8 sharp tip tends to have a slim central part while maltose sharp tip tends to have a thicker and stronger central part. Thus the force to make the needle with SU‐8 sharp tip bent is lower than that of the needle with maltose sharp tip. We also conducted the skin penetration tests with samples of different PDMS ratio. A 3 × 3 microneedle array was applied onto skin and the number of penetrated holes created by needles was recorded and the results are shown in Figure 2c and recorded in **Table** **1**. For the samples with a mix ratio of 1:4, there are 8 Figure 2i‐1 and 1 Figure 2i‐2 penetrated holes on skin for needles with SU‐8 sharp tips and maltose sharp tips respectively. For the samples with mix ratio of 1:6, there are 4 Figure 2ii‐1 and 3 Figure 2ii‐2 penetrated holes on skin for needles with SU‐8 sharp tips and maltose sharp tips, respectively. For the samples with a mix ratio of 1:8, there is no penetrated hole that can be found on skin for both needle configurations, as shown in Figure 2iii‐1 and Figure 2iii‐2. Thus, we could conclude that, in order to ensure a good skin penetration, the mix ratio of 1:4 is desirable for the device fabrication. The success penetration rate of the needle with maltose sharp tip is lower than that of needle with SU‐8 sharp tip when the test results of buckling force show an inverse trend. Although the maltose needle can stand a higher buckling force, it also requires higher force to penetrate the skin. This is because the thicker needle body of maltose sharp tip will affect a wider surface area during skin penetration, thus resulting in a higher possibility to get bent during skin penetration. Figure 2d shows the histology image of skin penetration by needles with SU‐8 sharp tip and maltose sharp tip. The microchannel created by the maltose sharp (Figure 2d‐i‐2) tip is broader than that of the SU‐8 sharp tip(Figure 2d‐i‐1), which confirms that the maltose sharp tip requires more force to penetrate skin. Another parameter that may affect the buckling force of the bendable microneedles is the angular of the PDMS pillars beneath the rigid sharp tips. When the angular of the pillars decreases from 60° to 30° and the angular of gaps between pillars increases from 30° to 60°, the anchor of the rigid sharp tip will take a higher ratio, making the needle more rigid and enhancing the buckling force. However, for the assembly of SU‐8 sharp tips by double drawing lithography as shown in Figure S2 (Supporting Information), the baking time to melt the SU‐8 tip assembled by the first step drawing cannot be well controlled to realize a partially filled gap for the samples with pillar angular lower than 55°. Because of the large gap angular, once melted, the SU‐8 always fills the whole gap, leaving no outlet for the drug to be delivered. Therefore, for the needles with SU‐8 sharp tips, we only use the pillars of 60° pillar angular. The drawing process to assemble maltose sharp tip is not limited by the pillar angular, thus the buckling force of needle with maltose needle by changing the pillar angular is evaluated and reported in Figure 2f. For each pillar angular, nine needles were tested. When the pillar angular decreases from 60° to 30° (Figure 2e), the buckling force of the individual needle increases from 0.43 N to 0.92 N because the whole microneedle becomes more rigid. However, the contact area between maltose and PDMS pillars also decreases with the decrease of the pillar angular. Thus the maltose sharp tip cannot be well fixed within the PDMS pillars and tends to break or detach from the PDMS pillars when the vertical force is applied to make the microneedle bent. For the case of 30° pillar angular, eight of nine needles showed sharp tip breakage. The possibility of the needle breakage decreases with the increase of pillar angular. For the needles of pillar angular more than 50°, no needle breakage was observed in the buckling force tests. As a conclusion, to avoid needle breakage and have buckling force as high as possible, pillar angular of 50° is the optimum value for microneedles with maltose sharp tips.

**Figure 2:**
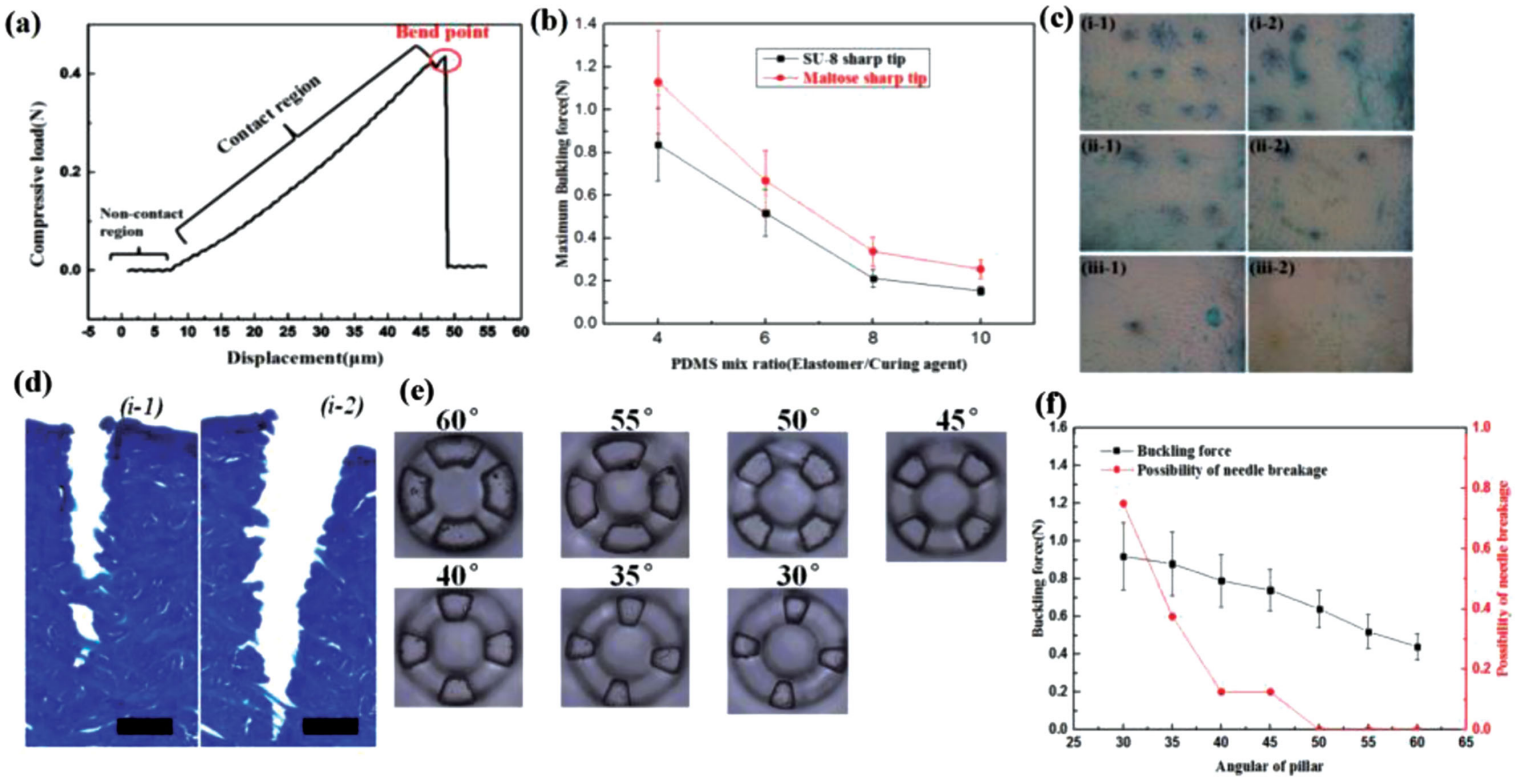
Optimization of PDMS stiffness and pillar angular of bendable microneedles for higher buckling force and success rate of skin penetration. a) A representative example of the buckling force test result for bendable microneedle; b) A representative example of the buckling force test result for bendable microneedle; c) Skin penetration results of microneedle with PDMS mix ratio of 4:1, 6:1, and 8:1 for needles with SU‐8((i‐1), (ii‐1) and (iii‐1)) and maltose((i‐2), (ii‐2), and (iii‐2)) sharp tips: (i‐1) and (i‐2): The mix ratio is 4:1 and the number of penetrated holes are 8 and 6, respectively; (ii‐1) and (ii‐2): The mix ratio is 6:1 and the number of penetrated holes are 4 and 3, respectively; (iii‐1) and (iii‐2): The mix ratio is 8:1 and there is no penetration holes on the skin; d) Histology image of skin penetration by needles with SU‐8 sharp tip(i‐1) and maltose sharp tip(i‐2). The scale bar is 200 μm; e) Optical image of the PDMS pillar of angular changing from 60° to 30°; f) Buckling force test and possibility of needle breakage of needle with maltose sharp tip by changing the pillar angular from 60° to 30°.

**Table 1:** Details of success rate of skin penetration for needles with SU‐8 and maltose sharp tips when the mix ratio of PDMS changes from 1:4 to 1:10

Mix ratio needle type	1:4	1:6	1:8	1:10
SU‐8 sharp tips	8/9	4/9	0/9	0/9
Maltose sharp tips	6/9	2/9	0/9	0/9

The dry adhesive film in the skin patch is used to fix the whole device onto skin surface and also provides the pulling force to separate the TEH patch from the skin, which will be mentioned in the next section. The detailed fabrication process can be found in Figure S1 (Supporting Information). To ensure the patch can be steadily fixed onto the skin, the normal adhesive force of the dry adhesive patch with two sets of pillar (diameter of 11 μm and 13 μm) and different ratio of pillar spacing versus pillar diameter(range from 20/11 to 3) was characterized. The adhesion strength was measured up to 10 repeating cycles for each sample by Instron Microtester 5848 (Instron, USA). The sample was attached onto the glass or skin and the normal force was applied by the Microtester from the backside to peel the dry adhesive off. The maximum force was recorded as the adhesive force. **Figure** **3**a,b show the adhesive force on glass and skin of each cycle. The dry adhesive was cleaned with acetone after each cycle to prevent the contamination and recover the adhesive force. The sample with 20 μm pillar spacing can achieve higher adhesive force than the sample with 24 μm pillar spacing when the pillar diameter is 11 μm. For the test on the glass, the adhesive force does not have an obvious decline as shown in Figure 3a. However, for the test on the skin, there is a decline of the adhesive force when repeating the test cycles. This is because the deformable skin will cause more contamination on the mushroom top when the dry adhesive was pressed onto skin surface. Thus repeating the test cycles will accumulate the contamination of mushroom and further reduce the adhesive force. The adhesive force will recover after cleaning the dry adhesive patch with acetone.

**Figure 3:**
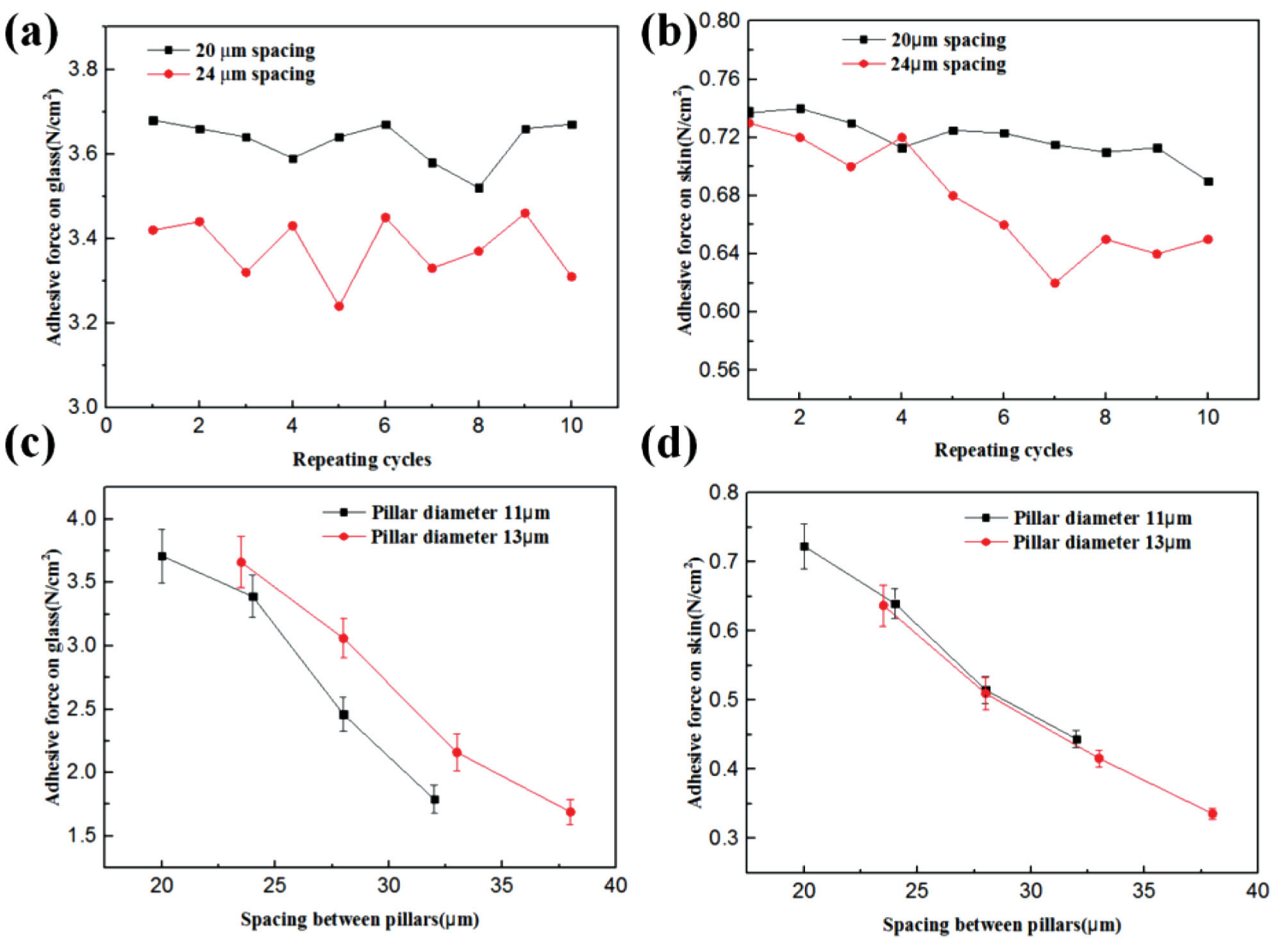
Characterization of the adhesive force of dry adhesive patches by changing the pillar diameter and pillar spacing. Adhesive force test by repeating 10 cycles on a) glass and on b) skin. After each test cycle, the dry adhesive patch was cleaned by acetone. The diameter of the pillar is 11 μm; Average adhesive force of the dry adhesive patches on c) glass and d) skin for samples with pillar diameter of 11 μm and 13 μm.

The characterization of the adhesive force on the glass and skin by changing the pillar spacing for pillars of 11 μm and 13 μm diameter is shown in Figure 3c,d. The test results agree with the data in (a) and (b). The adhesive force decreases when the spacing increases. And the adhesive force of the patch with 13 μm diameter is a bit lower than that with 11 μm due to the lower pillar aspect ratio when the pillar diameter is higher. Base on the characterization of the dry adhesive force, we can conclude that the sample with 11 μm pillar diameter and 20 μm pillar spacing can achieve the highest adhesive force. Therefore, this parameter is adopted for the dry adhesive to be integrated on the patch of complete device.

To integrate a power source on the thin flexible skin patch for active components which may be integrated in the future, a triboelectric patch was assembled. The detailed layer structure and fabrication process is shown in Figure S1 (Supporting Information).

A dielectric PDMS layer with micropatterns was used to enhance the effective surface area and output voltage. Here we studied the performance by using micropillar with and without mushroom top and pyramid as the micro‐patterns. At the backside of the dielectric PDMS layer, a Cu layer of 200 nm thickness was deposited by thermal evaporation. Then a Kapton layer was attached above the Cu layer to protect the metal from scratching and friction. As mentioned before, to adapt the different locations for our patch to be applied, we developed two methods to generate power. For the first case when the patch is applied on flat skin as shown in **Figure** **4**a, the distance between two dry adhesive patches is a bit shorter than the original length of the TEH patch, making the TEH patch bent upward and having no contact with skin surface (Figure 4a‐1‐i). Then the TEH patch is pressed by finger and contact with the skin surface, as shown in Figure 4a‐1‐ii, then is separated from skin again by lifting up the finger as shown in Figure 4a‐1‐iii. Due to the sticky surface of PDMS, once the TEH is attached onto the skin, it cannot separate from skin without a pulling force. Thus, a dry adhesive patch is assembled at the backside of the TEH patch, providing the pulling force by having the adhesive force between the finger and dry adhesive patch. The detailed charge transport process is shown in Figure 4a‐2. When the TEH patch was pressed onto the skin surface (Figure 4a‐2‐i), there is charge transport between them. According to the triboelectric theory, electrons are transferred from the PDMS to the skin during the electrification process, since PDMS is more triboelectrically negative than skin as shown in Figure 4a‐2‐ii. The change of the negative charges on the surface of the PDMS induces positive charges on the Cu electrode, driving free electrons to flow from the Cu layer to the ground. An output voltage or signal is generated. Once the finger is lifted up, making the PDMS and skin separated (Figure 4a‐2‐iii), the recovery of the surface negative charge on PDMS surface will induce a backflow of electrons from ground to Cu electrode. An opposite signal will be generated.

**Figure 4:**
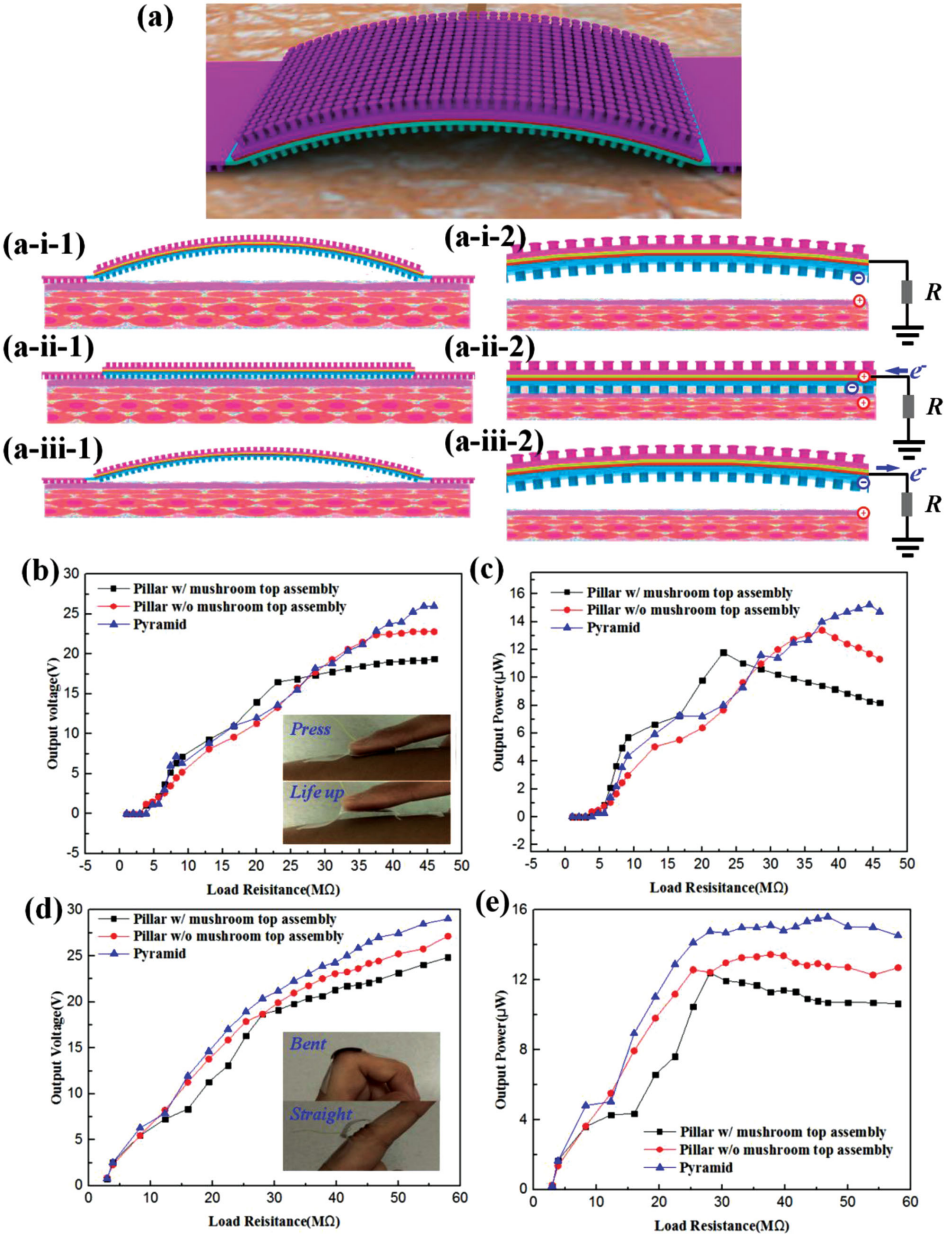
a) Working principle of the TEH patch when applied on flat skin surface; Characterization for the b) TEH output voltage and c) power when applied on flat skin; Characterization for d) the TEH output voltage and e) power when applied on finger knuckle.

The TEH is working in a contact‐mode and follows the Q‐V‐x relationship which is described in supplementary S4 (Supporting Information). To achieve the maximum output power, the TEH should be lifted up as high as possible. The maximum height to be lifted up is determined by the spacing between the two adhesives. When this spacing decreases, the TEH can bend upward more and have a large distance away from the skin surface. However, a shorter spacing between the two dry adhesive patches also decreases the effective contact area between the TEH patch and skin. It will further lower the quantity of the tribo‐charges to be generated. The spacing between the two dry adhesive patches is optimized in Figure S4 (Supporting Information) to achieve a maximum lift‐up height. The adhesive force should provide a certain adhesive force to lift up the TEH to the maximum height. Otherwise, before the TEH is lifted up to the maximum height, the finger will detach from the dry adhesive because of the insufficient pull force. Based on the adhesive force characterization in Figure 3, output voltage of dry adhesive patches with different adhesive forces are tested in Figure S4 (Supporting Information). The dry adhesive with highest output voltage was selected to be assembled at the backside of the TEH for output power characterization in Figure 4b,c.

In order to calculate the power generated by the TEH, a load resistor was connected between TEH patch and ground. The finger pressing of 2 Hz frequency was applied to generate the power. The voltage was then measured across the load resistor to obtain the power generated by the TEH. As the load resistance was increased, the power output increased, peaked at a point, and then started dropping thereafter. The voltage and power characteristics of TEH with different surface micropatterns and load resistance are shown in **Figure** **5**b,c. The maximum power output for TEH patch with pillar with and without mushroom top assembly and pyramid are 11.79, 13.3, and 15.21 μW, respectively; when the load resistance values are ≈23.08, ≈37.5, and ≈44.44 MΩ, respectively, as shown in Figure 4c. This load resistance for peak output power represents the inner impedance of the TEH patch. The TEH with pyramid surface micropatterns generates a higher voltage and output power, but also gives a higher inner impedance. A detailed analysis for explaining this difference is shown in supplementary S5 (Supporting Information).

**Figure 5:**
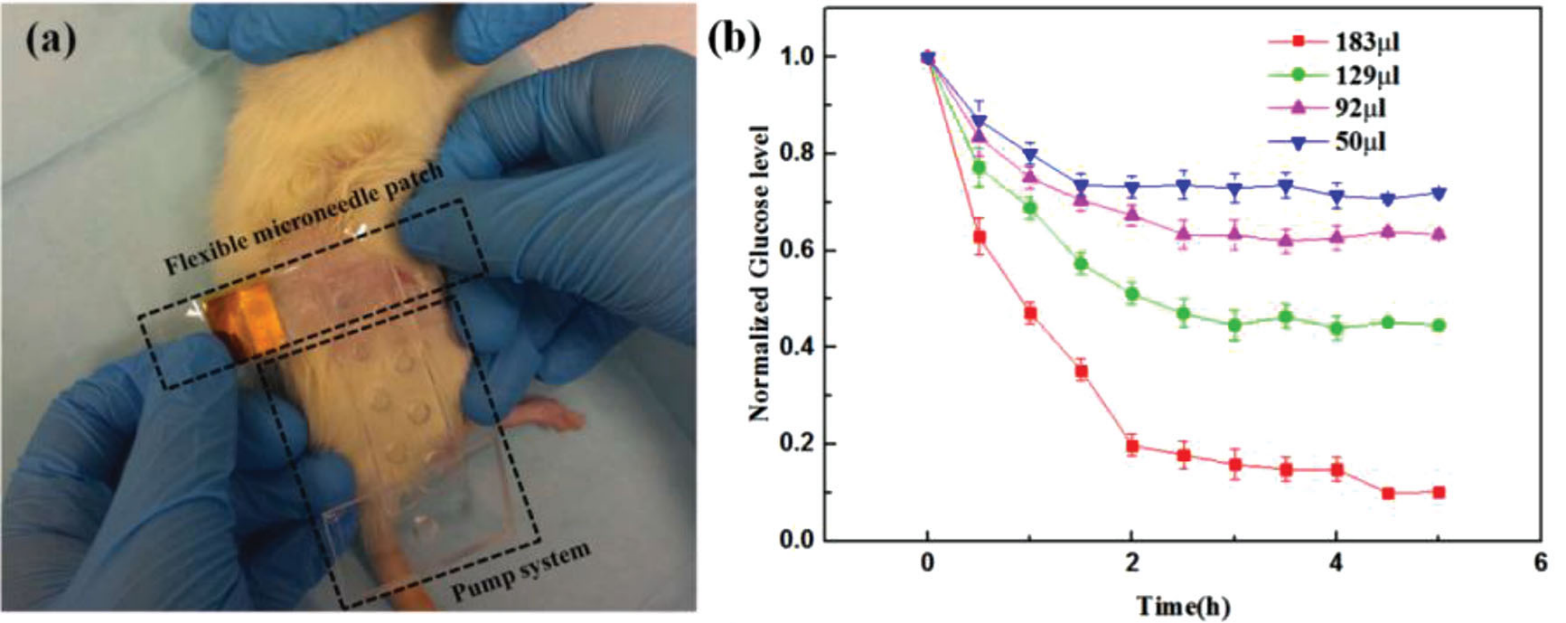
a) Image shows the flexible microneedle skin patch applied on the leg of a rat;b) Changes in blood glucose level in diabetic rats after insulin delivery using microneedles with different delivery volumes.

Another method to generate the power is to attach the device to the joint. In this case, when people bend and straight their joint, the skin will be stretched and compressed, making the TEH patch contact and detach the skin. Thus, the dry adhesive on the backside of TEH patch is not required. The test result of the output voltage and output power is shown in Figure 4d,e showing the same trend as that of (b) and (c). The maximum power output for TEH patch with pillar with and without mushroom top assembly and pyramid are 12.4, 13.4, and 15.6 μW, respectively; when the load resistance values are ≈28.1, ≈37.7, and ≈46.8 MΩ, respectively.

In order to confirm that the device has ideal features for an efficient drug delivery function, transdermal delivery of insulin was tested in vivo to prove the function by using the patch with SU‐8 sharp tips assembly in practical experiments.

The results are shown in Figure 5b. The blood glucose level in rats treated with our fabricated microneedles dropped continuously during the 5.5 h insulin delivery period and was quite stable after 3 h.

During the test with microneedle patches, the delivery volume can be controlled by controlling the pressing force. The delivered volume was recorded by measuring the weight to confirm the different volume delivered during the tests, as shown in Figure 5b. There were four groups in the study. For the first two groups, only one time pressing was applied during the test. The delivery volume was 50 μL and 90 μL, respectively. For the last two groups, the pump chambers were pressed twice and the delivery volume sensor was measured as 129 μL and 183 μL, respectively. The change of the blood glucose level is shown in Figure 5b. For the group with higher delivery volume, the blood glucose level dropped more. However, for all the groups, the blood glucose level stabilized at a certain level after 3 h. The experiment confirms that the manual control delivery mechanism with pump system can successfully control insulin delivery and further control the blood glucose levels.

In conclusion, we developed a stretchable wearable flexible medical device for transdermal drug delivery. Unique bendable microneedles were proposed to overcome the safety issue associated with the microneedle breakage during the application. The PDMS mix ratio and pillar spacing are optimized for a maximum buckling force to enhance the skin penetration success rate. To adapt the applications for water‐soluble and lipophilic drug formulations, two kinds of microneedle configurations are developed. Microneedles with SU‐8 sharp tips are more suitable for the storage of water‐based drugs while microneedles with maltose sharp tips are more suitable for the storage of lipid‐based drugs. We leverage the dry adhesive as the fixation method to realize a conformal attachment. The micropillar structure achieved in fabrication of dry adhesive is also investigated as a TEH patch, which is a promising energy source for other active components. Two methods for power generation, based on different positions for skin patch to be attached, were developed and characterized. Thus the TEH patch can generate power no matter the patch is attached on flat skin or joint part. The drug delivery function by using the bendable microneedle and pump system is confirmed by in vivo experiments in rats.

## Experimental Section

*Procedure of In Vivo Insulin Delivery Test*: All the procedures were performed under protocol and approved by the Institutional Animal Care and Use Committee at the National University of Singapore. Sprague–Dawley rats with a weight of 200–250 g were injected with 50 mg kg^−1^ streptozotocine (Sigma–Aldrich, Singapore) in citrate buffer (pH 4.2) via intraperitoneal injection to generate a diabetic animal model. These rats were kept with free access to food and water for 3 d. Then their blood glucose level was checked by a glucometer (Accu‐Chek, USA). The rats with a blood glucose level between 16 × 10^−3^ and 30 × 10^−3^ m were selected and the hairs on the abdomen skin were removed with a razor 24 h before the experiment.

After the rats were anesthetized, the fabricated microneedle patch skin patch with insulin loaded was applied on the abdomen skin surface. The pump chamber was pressed to deliver insulin (10 IU mL^−1^) of different volume from the drug reservoirs.

Blood samples were taken from the tail vein every 30 min after the beginning of the experiments in all groups. The blood glucose level monitoring lasted for 5.5 h. A glucometer (Accu‐Chek, USA) was used to give the corresponding blood glucose levels.

## Supporting information

As a service to our authors and readers, this journal provides supporting information supplied by the authors. Such materials are peer reviewed and may be re‐organized for online delivery, but are not copy‐edited or typeset. Technical support issues arising from supporting information (other than missing files) should be addressed to the authors.

Supplementary
