# Supplementary material for: Toward Self‐Powered Wearable Adhesive Skin Patch with Bendable Microneedle Array for Transdermal Drug Delivery
Source: Adv Sci (Weinh). 2016 Apr 19;3(9):1500441. doi: 10.1002/advs.201500441 (PMC5039970; doi:10.1002/advs.201500441)
Supplement: Supplementary file 1 — Supplementary [file ADVS-3-0c-s001.pdf]

Copyright WILEY-VCH Verlag GmbH & Co. KGaA, 69469 Weinheim, Germany, 2016.

Supporting Information

for *Adv. Sci.,* DOI: 10.1002/advs.201500441

Toward Self-Powered Wearable Adhesive Skin Patch with Bendable Microneedle Array for Transdermal Drug Delivery

*Hao Wang, Giorgia Pastorin, and Chengkuo Lee**

Stretchable Self-powered Wearable Adhesive Skin Patch with Bendable Microneedle Array
Supplementary information

S1. Fabrication process of the flexible skin patch

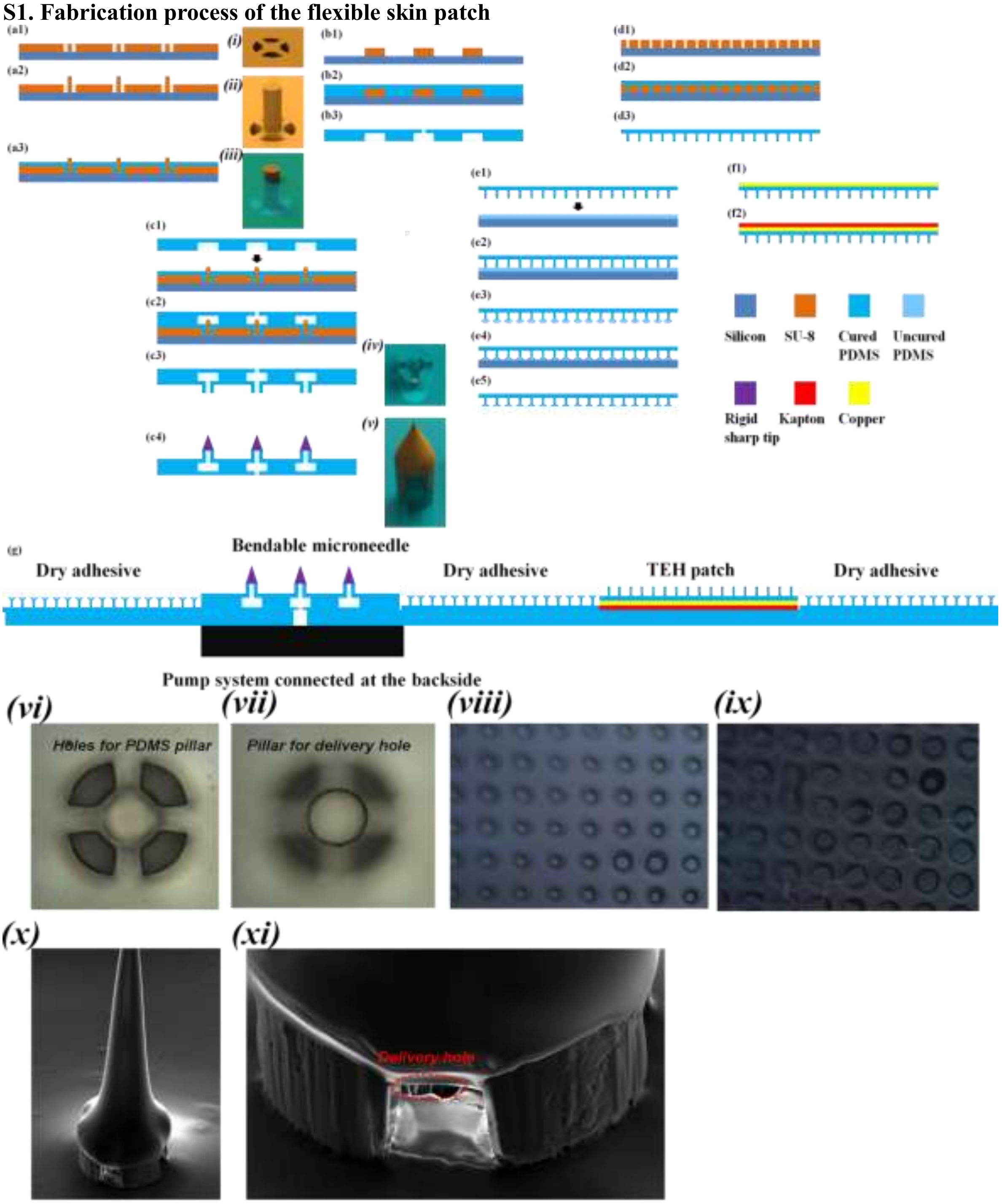

Fig. S1: The fabrication process of the flexible microneedle skin patch.

The whole patch consists of 4 functional components as shown in (g): bendable microneedle patch, dry adhesive, TEH patch and pump system connected to bendable microneedle patch for controlling the drug delivery.

1.1. Fabrication process for bendable microneedle array

(a), (b) and (c) show the fabrication process of the bendable microneedle patch. This microneedle comprises two PDMS layers and sharp tips.

The first PDMS layer is for the pillars supporting the sharp tips and the film holding the PDMS pillars as shown from (a1) to (a3). A SU-8 layer on silicon substrate was patterned to form the holes which are used as the mould for PDMS pillars as shown (i) and (vi). A SU-8 pillar was further patterned at the center of the holes as shown in (a2), (ii) and (vii). A PDMS layer was then spin coated onto the SU-8 layer to fill the SU-8 holes. But the thickness was not thick enough to cover the top of the SU-8 pillar as shown in (a3) and (iii). Thus, after the release of the PDMS layer off from the SU-8 layer, there will be a delivery hole located at the center of the four PDMS pillars as shown in (iv). These delivery holes form the connection between microneedles and other microfluidic channels.

The second layer is for the micro-channels array connecting the delivery holes as shown from (b1) to (b3). A SU-8 layer of the negative pattern of the micro-channel array was patterned on silicon substrate as shown in (b1) then covered with a PDMS layer as shown in (b2). Then the PDMS was cured by baking and released from the SU-8 layer as shown in (b3). A center hole was drilled by punch at the center of the channel array. This hole is to connect the channel array and the pump system in the final assembly process.

Then align the second PDMS layer with the first layer of PDMS which is still on the SU-8 mould as show in (c1) then bond these two layers by oxygen plasma treatment as shown in (c2). Release these two layers together off from the SU-8 mould as shown in (c3). Then rigid sharp tips were assembled by double drawing lithography as shown in (c4), (v), (x). (xi)

shows the delivery hole within the four-beam pillar structure. The material can be maltose or SU-8 by leveraging the similar process.

1.2. Fabrication process for dry adhesive

The dry adhesive is fabricated by leveraging the inking and printing technology. A PDMS layer with un-inked micro-pillar array was achieved by demoulding the PDMS from SU-8 mould as shown from (d1) to (d3). A SU-8 mould of 20μm thickness with micro-pattern of hole array was patterned on silicon substrate as shown in (d1). Then a PDMS layer was spun onto the SU-8 mould to fill all the holes on SU-8 mould as shown in (d2). Then cure and demould the PDMS layer off from the SU-8 mould as shown in (d3) and (viii). The height of the pillar is 20μm, which is the same as the thickness of the SU-8 mould.

The mushroom top which is necessary for dry adhesive to enhance the adhesive force is achieved by inking and printing process as shown from (e1) to (e5). A thin film of un-cured PDMS (5μm) was spun coat on a silicon chip as shown in (e1). Then the array of un-inked micropillars is inked in the un-cured PDMS film as shown in (e2). Subsequently, when lift the PDMS layer up from the un-cured PDMS film, small drops of un-cured PDMS is placed at the top of the array of un-inked micropillars as shown in (e3). Then the array was gently pressed against a silicon chip with treatment of detergent on the surface to peel sample easily as shown in (e4). Bake the sample to cure the PDMS droplet and release the dry adhesive off from the silicon chip as shown in (e5) and (ix).

1.3. Fabrication process for TEH patch

We leverage the pillar array which is achieved in (d3) as the surface micro-pattern required for TEH patch. A copper layer (200nm) was deposited by thermal evaporation at the backside of the PDMS layer as shown in (f1). Then a kapton layer was attached above the copper layer to fix the metal wire and protect the metal from scratching as shown in (f2). To study the different of performance by using different surface micro-patterns, we also use the dry

adhesive in (e5) instead of the pillar array in (d3) for the TEH patch. As a comparison with normal triboelectric patch, the pyramid surface micro-pattern is also used for TEH patch.

1.4. Fabrication process of the complete patch

All the functional patches are assembled onto a long PDMS sheet (200μm thickness) as shown in (g). For bendable microneedle patch, dry adhesive and pump system, they can be directly bonded onto the PDMS sheet by oxygen plasma treatment. Before the bonding of the bendable microneedle patch, a hole was drilled and aligned to the hole on the backside of the bendable microneedle patch to connect the bendable microneedle patch and pump system. The TEH is fixed by double side tape onto the PDMS sheet because the backside of the TEH patch is kapton and cannot be directly bonded by oxygen plasma.

1.5. Double drawing lithography to assemble microneedle array

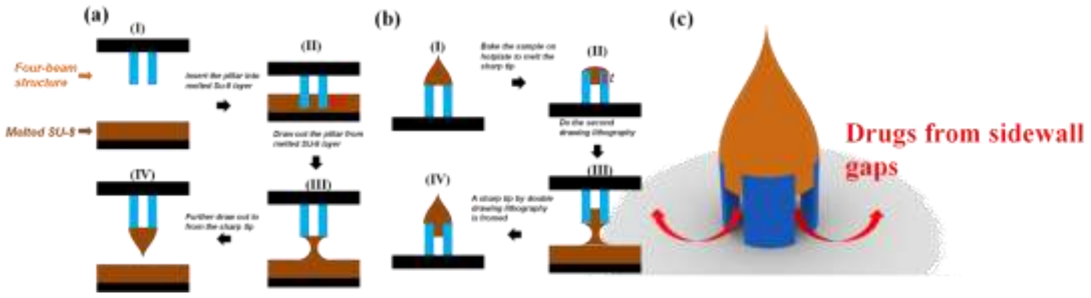

Fig. S2 Double drawing lithography for assembly of microneedle array

The process for double drawing lithography to assemble SU-8 sharp tips onto bendable microneedles is as shown is Fig S2. A pre-baked SU-8 layer was prepared on Si substrate. Then mount the sample above the SU-8 layer and bake the SU-8 layer to make it molten as shown in step (aI). Then insert the pillar into the melted SU-8 layer to a depth d as shown in step(aII). Draw out the pillar from the molten SU-8 layer. Some Su-8 will attach onto the top of pillar and a Su-8 bridge will be formed between the molten SU-8 layer and pillar as shown in step(aIII). Further draw out the pillar to break the SU-8 bridge and form the sharp tip as shown in step(aIV).

Then the whole device was baked in an oven at 120ºC to melt the hollowed SU-8 tips as shown in step(bII). Molten SU-8 reflowed into the gaps between four-beam sidewalls and the tips became domes. Then a second drawing process was conducted on the top of molten SU-8 to form sharp and solid tips as shown in step(bIII) and step(bIV). The flowing depth t of the melted SU-8 in the gaps could be controlled by changing the baking time in the reflow step. Due to the pillar for drawing lithography is a four-beam structure, which means there are gaps along the sidewalls. Thus the drug could flow out the microneedle from the gaps along the sidewall as shown in Fig. S2(c).

S2. Structure and working principle of the pump and check-valves for microfluidic control system

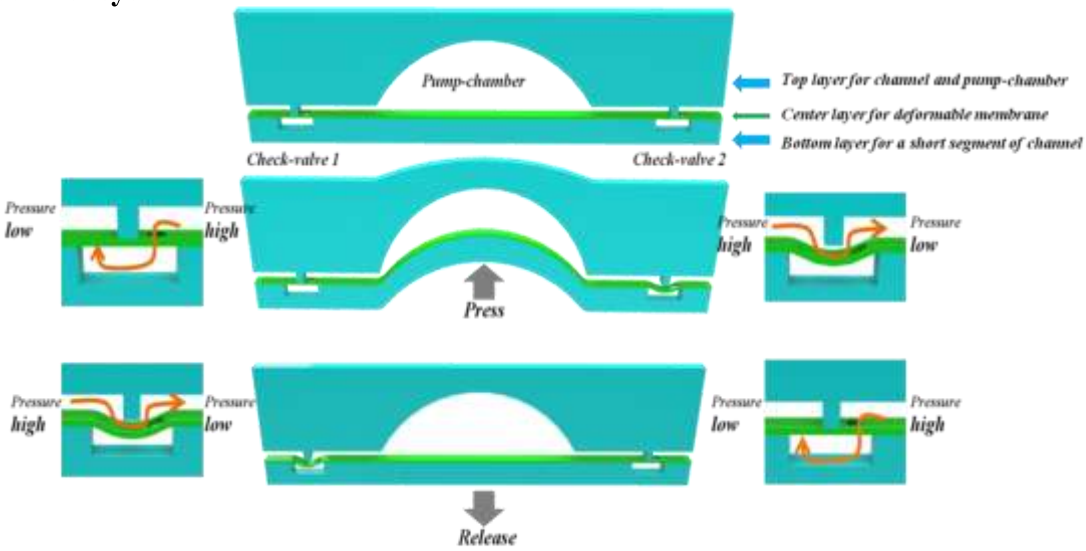

Fig. S3 Structure and working principle of the pump chamber and check-valves.

The design and working principle of the pump-chamber and check-valves are illustrated in Fig. S3. The pump -chamber is located between two check-valves. The check-valve has three parts: a thin PDMS membrane layer with a hole, a top layer that contains a discontinuous channel, and a bottom layer that contains a short segment of a channel. The device was formed by bonding these three layers together. But the contact area between the protruding poster of the top layer and center membrane layer should not be treated with Oxygen plasma. When pressure applied, the center membrane could detach from the top layer and deform.

During the plasma treatment, the contact area will be covered by a piece of Al foil. These two check-valves ensure that the liquid or air can only enter the pump-chamber through one valve, and leave the pump-chamber through the other. Due to the elasticity of PDMS, when the bottom layer of the pump-chamber is pressed and deformed, the pressure in the pump-chamber will increase. For check-valve 1, this high pressure in the pump-chamber will make the chamber below the membrane expand. Thus the membrane will be pushed and attach tightly onto the protruding poster of the upper layer, sealing the channel. Then check-valve 1 is off. For check-valve 2, this high pressure in pump-chamber will push the membrane of the check-valve 2 and make it deform. Then check-valve 2 is on and the air or liquid in the pump chamber can pass through. When the bottom layer of the pump-chamber is released, the pump-chamber tends to recover its initial shape. Thus the pressure in pump-chamber is low. This low pressure in the pump-chamber will make the membrane of check-valve 1 have a downwards deformation. Then the check-valve 1 is on and the air or liquid outside the device can be sucked into the pump-chamber. Meanwhile the low pressure in pump-chamber will make the membrane of check-valve 2 have upwards deformation and seal the channel. Then check-valve 2 is off. In summary, one check-valve can be on only when another check-valve is off. Then a one directional flow can be formed by pressing the pump chamber.

S3. Optimization for TEH patch configuration

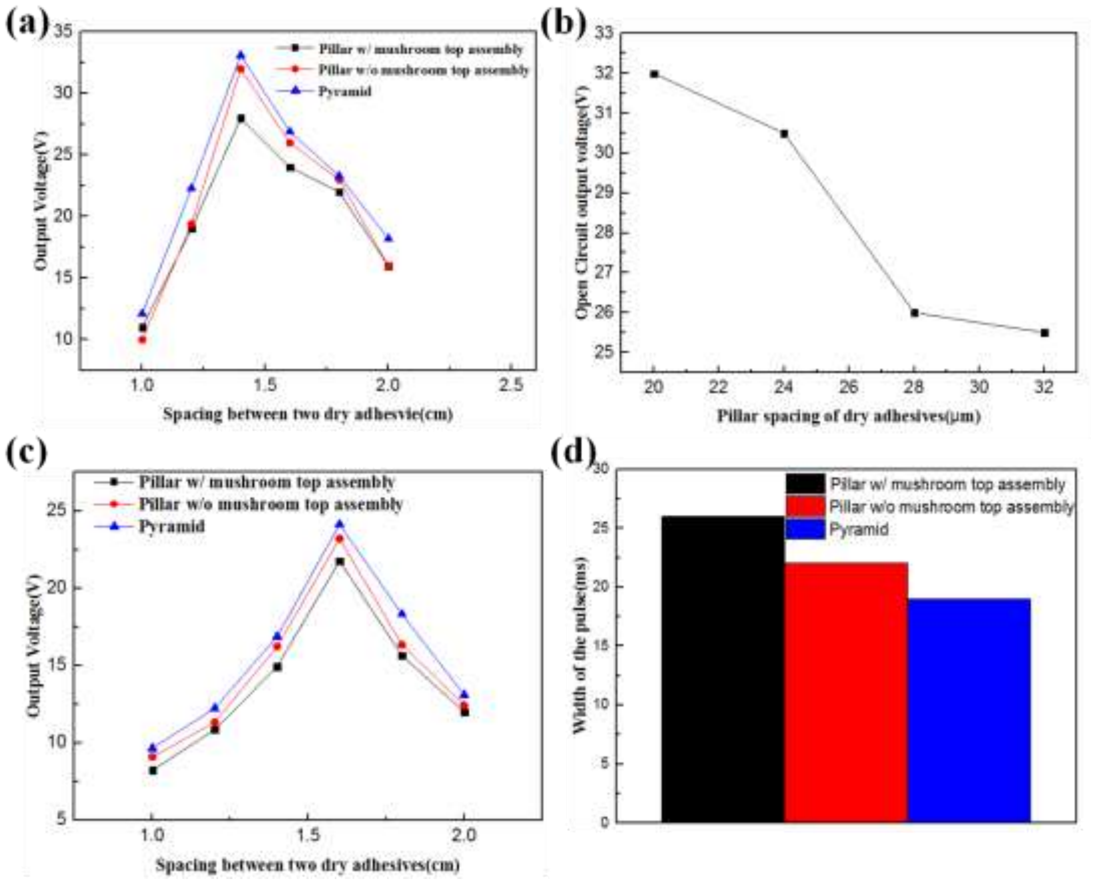

Fig. S4 (a) Open circuit voltage by changing the spacing between two adhesive for the TEH attached on arm; (b) Open circuit voltage by changing the pillar spacing of the dry adhesive patch at the backside of the TEH patch; (c) Open circuit voltage by changing the spacing between two adhesive for the TEH attached on finger knuckle; (d) Average width of the pulse for the signal in (a).

The spacing between the two dry adhesive patches beside the TEH patch will affect the maximum height to be lifted up and further affect the maximum output voltage. The test results of the output voltage of the TEH patch by changing the spacing between two dry adhesive patches for the situation when the TEH was applied on arm is shown in (Fig S4(a)). The length of the TEH patch is 2cm. Thus the spacing between two dry adhesive patches decreases from 2 cm to 1cm with 0.2cm step. The output voltage peaks at 1.4cm because the increase of the height to be lifted up. Then it declines by reducing the spacing because the effective contact area reduces when the spacing is too low. For the situation when the TEH patch was applied on finger knuckle, the rest results are shown in Fig. S4(c). The output voltage peaks at 1.6cm. Another fact will affect the lift- up height is the adhesive force of the

dry adhesive patch assembled at the backside of the TEH patch. If the adhesive force is not sufficient, it will detach from finger before the TEH patch is lifted up to the maximum height. Based on the adhesive force characterization in Fig. 4, we selected the sample of 11μm and pillar spacing ranges from 20μm to 32μm in the test. In this test, only micro-pillar without mushroom top is used. As shown in Fig. S4(b), the voltage decrease with the increase of the pillar spacing because of the reduction of the adhesive force. Only the dry adhesive patch with 20μm spacing can give the highest output voltage which indicates that the highest lift-up height. The width of the pulse for the signal measured in Fig. S4(a) is shown in Fig. S4(d). As mentioned in paper, because the TEH patch with pillar-based micro-pattern is stickier than the that with pyramid micro-pattern, thus it takes longer time to detach from skin surface and generate a broader pulse. The average width of the pulse for pyramid, pillar with and without mushroom top assembly is 19ms, 22ms and 26 ms, respectively.

S4. V-Q-x relationship for contact mode TEHs

According to the V–Q–x relationship for contact-mode TEHs [1], the output voltage is determined by the following equation:

$$V = E_{dielectric} \times d + E_{air} \times x \quad (1)$$

where $E_{dielectric}$ is the electric field though the dielectric layer, which is PDMS layer here, generated by the tribo-charges on the opposite sides of the TEH; d is the thickness of the dielectric layer; E_{air} is the electric field though the spacing between the top surface of TEH and contact surface, this electric field is generated by the tribo-charges on the TEH surface and contact surface; x is the spacing between the TEH surface and contact surface. The output voltage should increase with the increase of the spacing between the TEH surface and contact surface in the ideal fully contact-mold TEHs.

S5. Explanation for why TEH with pyramid surface micro-patterns generates a higher voltage and output power, but also gives a higher inner impedance.

The inner impedance is determined by the following equation:

$$R_{opt} \approx \frac{(d_0 + x_{\max})^2}{Sv\varepsilon_0} \quad (2)$$

where R_{opt} is the inner impedance; d_0 is the thickness of the PDMS layer; x_{max} is the maximum height to be lifted up for TEH patch; S is the effective area size of TEH patch which can be altered by the surface micro-patterns; v is the speed of the TEH to be lifted up; ε_0 is the dielectric constant of air.

For the TEH patches whose surface micro-patterns are pillar with and without mushroom tops, the contact surface is stickier than the TEH patches with pyramid surface micro-pattern. Thus when the TEH patches were lifted up, TEH with pyramid surface micro-pattern has a faster detachment from skin, thus with a higher v, result in a narrower and higher output voltage pulse. However, when the height of the micro-pillar, which is 20μm, is much higher than the height of the pyramid, which is 1.37μm, the micro-pillar structure can provide a higher S, effective area size of TEH patch, than the pyramid structure and further lower the inner impedance.

Reference

[1] Niu, Simiao, Sihong Wang, Long Lin, Ying Liu, Yu Sheng Zhou, Youfan Hu, and Zhong Lin Wang. "Theoretical study of contact-mode triboelectric nanogenerators as an effective power source." Energy & Environmental Science 6, no. 12 (2013): 3576-3583.
